# Direct Experimental Observation of the Tetrabromine Cluster Br_4_ by Synchrotron Photoionization Mass Spectrometry

**DOI:** 10.1002/open.202300266

**Published:** 2024-02-02

**Authors:** Rory McClish, Giovanni Meloni

**Affiliations:** ^1^ Department of Chemistry University of San Francisco San Francisco CA 94117 USA; ^2^ Department of Biological and Chemical Sciences New York Institute of Technology Old Westbury NY 11568 USA

**Keywords:** synchrotron photoionization, multiplexed mass spectrometry, adiabatic ionization energy, tetrabromine cluster, distorted tetrahedral *D_2h_* structure

## Abstract

We present a first spectroscopic characterization of the homoatomic polyhalogen tetrabromine, Br_4_, in the gas phase. Photolysis of CHBr_3_ at 248 nm is used to generate atomic bromine radicals in a flow tube reactor. Resulting combination products are detected by photoionization mass spectrometry at the Advanced Light Source of the Lawrence Berkeley National Laboratory. Interpretation of the experimental mass spectra is informed by calculated adiabatic ionization energies carried out at the CCSD(T)/aug‐cc‐pVTZ//M06‐2X/aug‐cc‐pVTZ and CCSD(T)/aug‐cc‐pVTZ//cam‐B3LYP/6‐311++g** levels of theory. Tunable VUV synchrotron radiation enables the collection of the mass‐selected photoionization spectra by which Br_4_ is assigned using Franck‐Condon simulations of a Br_2_ dimer with a stretched tetrahedral geometry.

## Graphical abstract

Pure halogens clusters, especially with even numbered molecular formulae, are elusive gas‐phase spectroscopic targets. Photoionization mass spectrometry coupled to tunable vacuum‐ultraviolet synchrotron radiation allows for the detection and characterization of the tetrabromine Br_4_ cluster. Based on a joint experimental and computational assessment, Br_4_ is found to exist as a tetrahedron shape of *D_2h_* symmetry.

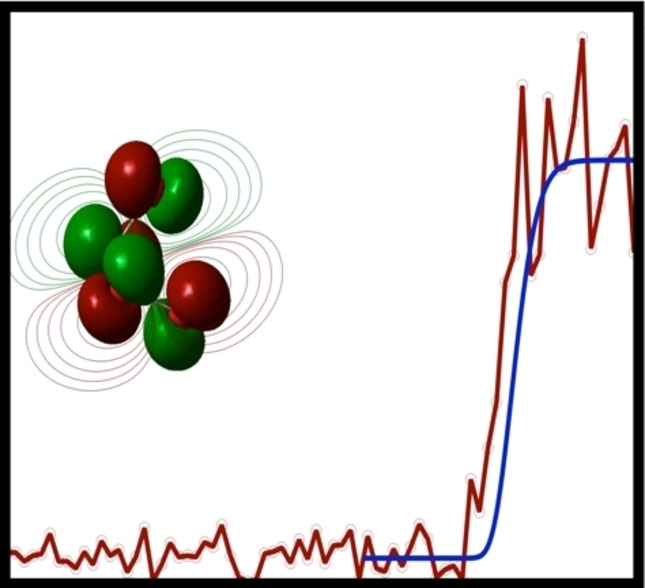

## Introduction

Homopolyatomic halogen chemistry has a rich historical foundation since the late 18^th^ century and continued interest today.^[1]^ Fundamental insights into such chemistry is driven by a wide range of phenomena. Polybromine crystals have use in superconducting materials, whereas atomic and molecular bromine have a significant role in climate cycles including ozone depletion.^[2]^ Although diatomic halogens have long been well characterized, this renewed interest expands to larger clusters, *X_n_*, for n>2.^[3]^ Triatomic clusters, *X_3_*, have been spectroscopically characterized for *X=*Cl, Br, and I by X‐ray crystallography and Raman spectroscopy.[^3c^, ^4^] Interaction of *X_3_* with the dimer, *X_2_*, is found to form *X_5_* for *X=*Br, I.^[5]^ Clusters with *n=*4 are more elusive. Gillespie *et al*.^[6]^ synthesized the diamagnetic I_4_^2+^ in the solid state (while Brichall and Myers^[7]^ found it in solution) with a structure described by π *– π * overlap and having a square planar geometry. Additionally, Chance *et. al*.^*[8]*^ conducted electric deflection studies on a set of halogen clusters and proposed that Br_4_ is polar. Multiple theoretical investigations have worked to further propose the structure of Br_4_. Schuster *et. al*.^*[9]*^ conducted a DFT study of neutral and ionic polybromine clusters from *n=*1‐5 and proposed a L‐shaped neutral Br_4_ and a twisted Br_4_^+^ with *C_s_* and *C_1_* symmetry, respectively. However, Thanthiriwatte *et. al*.^*[10]*^ included dispersion corrected DFT methods as well as *ab‐initio* CCSD(T) to argue that Br_4_^+^ has a planar *D_2h_* symmetry. Long range intermolecular interactions are certainly expected to play a role, but thus far theory has not been able to be compared to experiment.

Photoionization mass spectrometry coupled to tunable VUV light offers a compelling opportunity to detect and characterize the tetratomic bromine cluster. The equilibrium structures of both neutral and cationic Br_4_ is expected to determine the ionization energies with isomeric selectivity. Moreover, by tuning the photon energy of the ionizing synchrotron radiation over the first ionization potential of a species, the shape of the resultant photoionization (PI) spectrum is dependent on the photoionization cross section. The cross section itself is partially dependent on the transition between neutral and cation, thereby further reporting on the structure of a target. We herein present measurement of the Br_4_^+^ cation through the use of photoionization mass spectrometry (PIMS) via tunable synchrotron radiation. Structural assignments are rationalized via quantum chemistry calculations and comparison of integrated theoretical photoelectron simulations.

## Experimental and Computational Methods

Experiments were carried out at the Advanced Light Source (ALS) of Lawrence Berkeley National Laboratory. A more detailed explanation of the experimental technique is available and only a brief summary is given here.^[11]^ Dilute bromoform (CHBr_3_, 1 % in Ar) was flowed into a vertical 62 cm long quartz flow‐tube reactor (~1 cm I.D.) and subsequently photolyzed by a pulsed KrF excimer laser at 248 nm fired at 10 Hz and 30 kV. The laser power was measured at 406 mJ/pulse and the measured power transmittance through the reactor tube was 17.5 %. Resulting bromine atoms are produced via the multiphoton dissociation of CHBr_3_. Estimating the number density of bromine radicals at this stage is unfeasible due to the five proposed photodissociation pathways of bromoform accessible in the UV region.^[12]^ Reaction species escape out sideways from the reactor via a 650 μm pinhole and the expansion is skimmed using a 1.5 mm diameter skimmer. The molecular beam is orthogonally intercepted by tunable synchrotron light stepped at 25 meV between 8.00–10.75 eV. A shift of +0.0272 eV based on known Xenon resonances is applied after calibration and all spectra are normalized to the measured VUV photocurrent. Additionally, ion‐signals appearing post photolysis are background subtracted to show only the ion signal contribution after the excimer laser begins firing at t_rxn_=20 ms. Photoionization occurs if the photon energy exceeds the ionization energy of a neutral species in the molecular beam. For a given ionization event, each photoion born from the molecular beam is subject to focusing by static electric fields onto a multichannel plate detector (MCP). The resulting ion counts as a function of flight time thus compose a time‐of‐flight mass spectrum. The tunable synchrotron VUV light serves a crucial purpose for product characterization, as the collected ion signal is a function of mass, reaction time (determined by the triggering of a photolysis laser), and photon energy. Integrating over carefully selected mass and time bounds allows for plotting the ion signal as a function of photon energy to reveal a mass‐selected photoionization (PI) spectrum. Bromine‐containing peaks are quickly identified in the mass spectrum based on the 51/49 isotopic abundance between ^79^Br/^81^Br. Furthermore, since the shape of the PI spectrum is dependent on the difference between the neutral and cationic structures, the spectral onset is indicative of the ionization energy (IE) of the species, while the shape of the PI curve lends clues to the geometry of any observed species.

Electronic structure calculations were carried out on the *Gaussian 09* ^*[13]*^ computational chemistry software package. Two density functional theory (DFT) methods are selected for geometry optimizations and frequency calculations. The ubiquitous Becke three‐parameter hybrid functional variation B3LYP with the Coulomb Attenuating Method (cam‐B3LYP) is known to well account for long range interactions.^[14]^ The second functional used is the Minnesota functional M06‐2X, a global hybrid functional well parameterized for main group non‐metals with double the nonlocal exchange as M06.^[15]^ Frequency analysis is incorporated to confirm that each optimized structure is at a true local minimum on the potential energy surface. Both methods are used with the aug‐cc‐pVTZ basis set, i. e., Dunning's polarized correlation consistent triple‐zeta basis set augmented with diffuse functions.^[16]^ The optimized structures are fed to CCSD(T)/aug‐cc‐pVTZ single point calculations in order to refine the energetics of the system at a high level of theory.^[17]^ Relative energetics are calculated as the sum of the electronic energy found at CCSD(T) and the zero‐point energy from a given DFT. The CCSD(T) energies and DFT normal mode frequencies allow for calculation of the transition dipole moment and overlap integrals between neutrals and cations, which determines the progression of a simulated photoelectron spectrum (PES).^[18]^ These Franck‐Condon simulations are then integrated such that they are analogous to a photoionization spectrum.

## Results and Discussion

**Figure 1:**
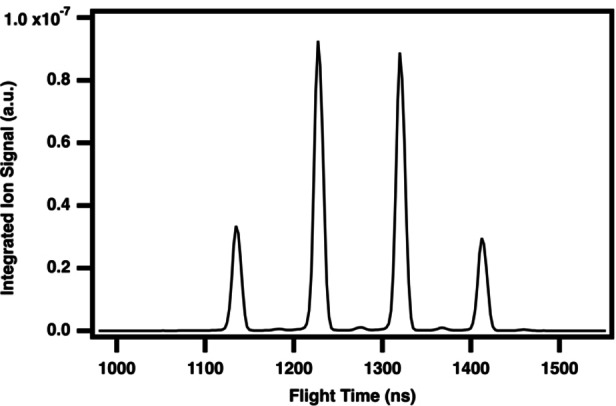
Photoionization time‐of‐flight mass spectrum of bromoform, CHBr_3_, integrated over 8.4–10.75 eV, 0–20 ms reaction time (pre‐photolysis), and normalized to the photocurrent.

Intense ion signals in the time‐of‐flight mass spectrum are observed at small flight times and constitute “turnaround” mass peaks, that is, ions that are heavy enough to have flight times so long that they appear early (or fast) in subsequent sweeps across the microchannel plate detector. The flight times of such turnaround ions are thus no longer meaningfully associated with the mass‐to‐charge ratio. This context arises in our study because the ion optics in the mass‐spectrometer were optimized for a separate investigation of the reactivity of the methylidyne radical (CH(X^2^Π) for which bromoform serves as a radical precursor) towards other organic hydrocarbons, where the following discussion of tetrabromine is thus an unexpected yet exciting observation. Consequently, the mass spectrum does not extend beyond m/z 159. Despite lacking the m/z value of a given turnaround peak, the tunable ALS synchrotron radiation allows to collect a dependable PI curve to use for spectroscopic characterization. We thus first demonstrate this capability by the characterization of the reactant bromoform, CHBr_3_, as a turnaround cluster of intense peaks appearing at nominal m/z ratios between 4.5–6 amu. This section of the TOF‐MS ion signal integrated across pre‐photolysis times and over the full photon energy range of 8.4‐10.75 eV is shown in Figure 1, in which the most intense cluster of peaks collected in our full dataset appears appear in a 1 : 3 : 3 : 1 ratio. This splitting in the time‐of‐flight is consistent with the isotopic abundance of a Br_*n*_ containing species of *n*=3. Additionally, small adjacent peaks mimicking the splitting but roughly 1.1 % in relative intensity to the intense peaks are interspersed between and are easily assigned as the contribution from the corresponding ^13^C‐containing isotope combinations.

**Figure 2:**
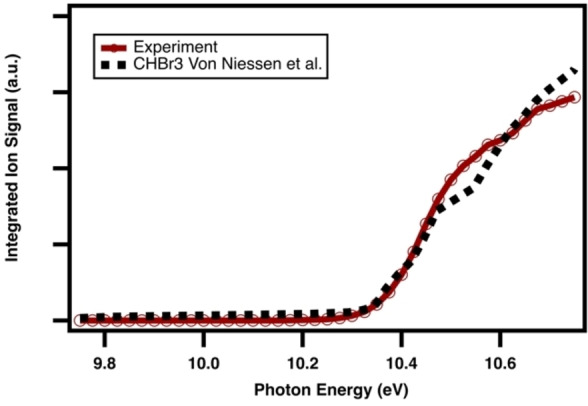
Observed mass‐selected photoionization spectrum of bromoform, CHBr_3_, integrated over flight times of 1100–1500 ns, 0–20 ms reaction time (pre‐photolysis), and normalized to the photocurrent (red with hollow circles). Reference photoelectron spectrum manually digitized and integrated from Von Niessen *et al*.^[19]^

Identical photoionization spectra (Figure 2) are obtained for each individual peak in the TOF spectrum, the sum of which can be appropriately obtained by integrating over the full flight‐time of the isotope cluster and the reaction time of 20 ms pre‐photolysis (before the excimer laser starts firing). The experimental curve finds good agreement with the known photoelectron spectrum^[19]^ of CHBr_3_, from which integration yields an analogous PI spectrum for comparison to our collected signal.

**Figure 3:**
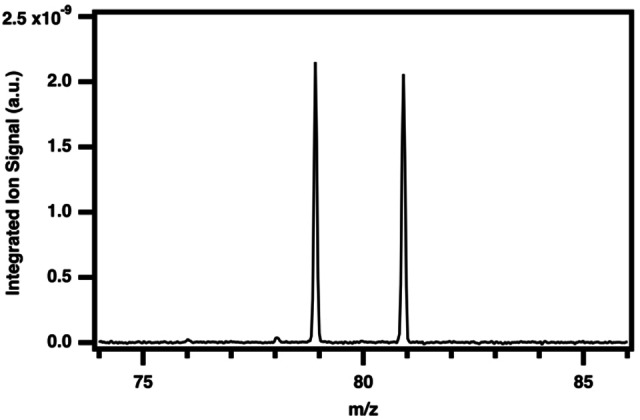
Photoionization time‐of‐flight mass spectrum of atomic bromine cation fragment, integrated over 8.4‐10.75 eV, 0–100 ms reaction time, background subtracted, and normalized to the photocurrent.

Upon triggering of the reaction via the excimer photolysis laser, evidence of brominated reaction products is observed in the mass spectrum. Characterization of Br_2_ is first achieved by identifying the peaks at m/z 79, 81, and 158. The range of the mass spectrum did not extend past m/z 159, and the isotopic peaks at m/z 160 (^79/81^Br_2_) and 162 (^81/81^Br_2_) are thus not observed. However, the isotopic distribution of Br_2_ peaks would be expected to have a relative intensity of approximately 1 : 2 : 1. Molecular bromine is additionally known to have an autoionization fragment ion Br^+^ (arising from an ion‐pair process) of expected 1 : 1 relative intensities for m/z 79 and 81, as seen in Figure 3.^[20]^ We observe an ion signal of m/z 158 near 10.5 eV, matching the known ionization energy of Br_2_ of 10.518 eV. The Br^+^ fragment peaks at m/z 79 and 81 appear near 10.33 eV, in agreement with previous measurements.[^20^, ^21^] Any contribution to the ion signal originating from neutral atomic bromine at m/z 79/81 can be ruled out as it has an IE of 10.81 eV, which is above the photon energy scanned in this study.^[22]^

**Figure 4:**
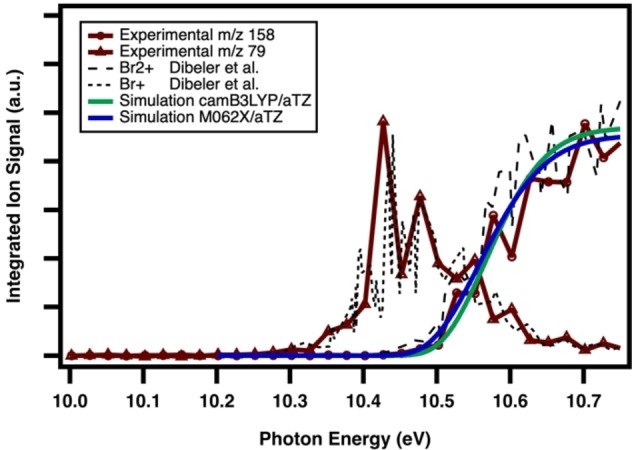
Observed mass‐selected photoionization spectrum of molecular bromine integrated and background subtracted over 0–100 ms reaction time, and normalized to the photocurrent. Red with hollow circles is the observed m/z 158 curve, and red with solid triangles is the m/z 79 fragment PI curve. Reference threshold photoelectron spectrum manually digitized and integrated from Dibeler et al^[23]^ of Br_2_ and autoionization fragment Br (dashed black lines). Simulation of Br_2_ at the camB3LYP/aug‐cc‐pVTZ (green) and M062X/aug‐cc‐pVTZ (blue) are superimposed onto as well.

Characterization is further verified by agreement between the respective photoionization spectra with literature plots in Figure 4.^[23]^ Additionally, the simulated photoelectron spectrum of Br_2_ is integrated and included as a first test of the choice of computational methodology. Benchmarking both the cam‐B3LYP and M06‐2X functionals with Br_2_ provides good agreement with established experimental results on structure and vibrational frequencies. Using the former method, error in the calculated Br−Br bond length is found to be 0.4 %, with a deviation of 4.4 % error from the established Br−Br stretch vibrational frequency, while for the Minnesota functional the percent errors are 0.2 % and 6.3 %, respectively. Single point energies are subsequently calculated at the *ab‐initio* level CCSD(T)/aug‐cc‐pVTZ. The calculated adiabatic ionization (AIE) energy is taken as the energy difference of the zero‐point corrected electronic energy of the cation and neutral ground state structures, respectively. Starting with either DFT functional before refining with CCSD(T) yields an AIE of 10.58 eV, which is 66 meV higher than the experimentally established IE of 10.52 eV by TPES.^[24]^

**Figure 5:**
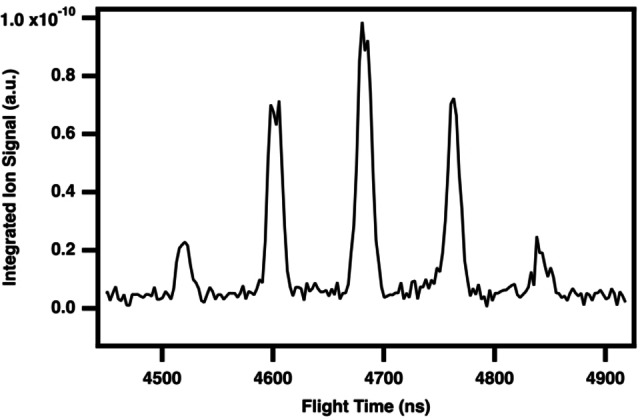
Photoionization time‐of‐flight spectrum of post‐photolysis reaction product integrated over 8.4–10.75 eV, 0–100 ms reaction time with background subtraction, and normalized to the photocurrent.

**Figure 6:**
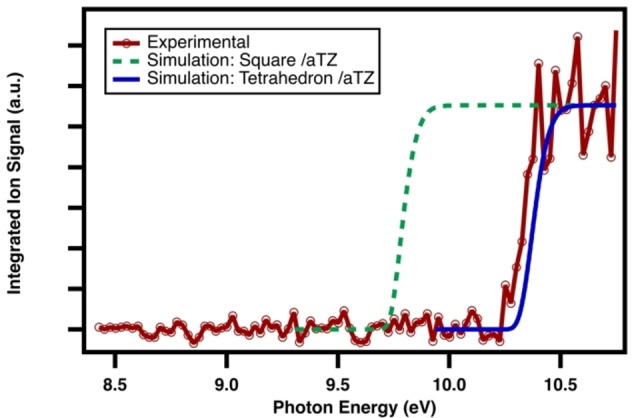
Observed mass‐selected photoionization spectrum integrated over flight times of 4500–4900 ns, 0–100 ms reaction time and background subtracted, and normalized to the photocurrent. Observed PI curve (red with hollow circles), simulation of “square” geometry at camB3LYP/aug‐cc‐pVTZ (dashed green), and simulation of stretched tetrahedron geometry at M062X/aug‐cc‐pVTZ (solid blue).

A cluster of intense peaks are observed in the m/z 20–23 range of the mass spectrum and only grow in as post‐photolysis signals. We believe these to be reaction products detected as turnaround peaks derived from two loosely tethered Br_2_ molecules. The ion signal originating from a bromine‐containing species of *n*=4 is hinted at through an observed isotopic distribution consistent with a corresponding expected 1 : 4 : 6 : 4 : 1 relative intensity of the integrated peak areas (Figure 5). Notably, in contrast to the CHBr_3_ signal, this cluster of peaks shows no indication of a ^13^C signal, and therefore is expected to be from a carbon free molecule with four bromine atoms. Identically shaped PI signals are obtained for each isotopic peak, and the onset of the curve is observed at 10.2 eV with a sharp step‐like transition. The PI spectrum's shape qualitatively indicates favorable Franck‐Condon factors and is shown in Figure 6. In the case of Br_4_, geometries are taken from both Schuster *et al*.^[9]^ and Thanthiriwatte *et al*.^[10]^ Upon re‐optimization of the *D_2h_* geometry at the cam‐B3LYP/aug‐cc‐pVTZ level, the cation shares the *D_2h_* symmetry of the neutral cluster but with contracted bond lengths between both the two Br_2_ subunits and within the Br_2_ units themselves. This “square” configuration of two Br_2_ subunits for both the neutral and cationic case is thus analogous to a homonuclear diatomic. Neither optimized structure possesses any calculated dipole moment, and both geometries are nonpolar. No successful optimization of the square *D_2h_* geometry was found with M06‐2X.

Conversely, starting with the L‐shaped neutral, in which one Br_2_ is colinear with one end of the other Br_2_, the cation equilibrates to a new configuration with the notable distortion being a break in symmetry from *C_s_* to *C_1_* based on a dihedral angle change of 90 degrees such that the internuclear axis between each Br_2_ moiety is approximately orthogonal. This is found to be the case using both functionals. Lastly, we consider the case of an elongated tetrahedral geometry not previously discussed in the literature. This local minimum is located with the M06‐2X functional but did not converge in attempts using cam‐B3LYP. Instead of two Br_2_ in a planar configuration, one is rotated 90°. However, while forming a tetrahedron, the Br_2_−Br_2_ distance is greater than each diatomic bond such that the symmetry is reduced from *T_d_* to *D_2h_*. A summary of the DFT optimized parameters, including geometries and harmonic vibrational frequencies, is provided in Table 1.

**Table 1:** Optimized parameters for neutral and cationic Br_4_ isomers: geometry labels; equilibrium bond lengths, R_e_, in Å; bond angles in degrees, α; dihedral angle in degrees, γ; vibrational frequencies in wavenumbers cm^−1^, ω.

CAM−B3LYP/aug‐cc‐pVTZ					
Geometry label	R_e_	ɑ	γ	ω	ZPE (meV)
L	2.297	101.52	–	4.83	48
	3.196			30.46	
	2.290			33.60	
				40.65	
				327.01	
				330.45	
L+	2.252	109.54	91.41	14.50	53
	3.017	109.54		33.56	
	2.252			36.59	
				104.15	
				329.68	
				340.10	
square	2.291	90.00	0.00	3.13	43
	4.817			4.35	
	4.817			10.04	
	2.291			12.21	
				333.14	
				333.26	
square+	2.239	90.00	0.00	30.47	57
	3.347			40.12	
	3.347			68.73	
	2.239			85.66	
				342.02	
				359.65	

M06‐2X/aug‐cc‐pVTZ					
Geometry label	R_e_	ɑ	γ	ω	ZPE (meV)
L	2.291	88.37	–	39.09	56
	3.365			56.18	
	2.284			59.27	
				71.38	
				334.96	
				342.22	
L+	2.246	105.92	93.63	32.14	59
	2.997	105.92		51.86	
	2.246			57.22	
				117.35	
				338.53	
				347.91	
tetrahedron	2.282	–	84.75	50.79	59
	3.920			69.08	
	3.920			72.94	
	3.920			73.00	
	3.921			343.58	
	2.282			344.16	
tetrahedron+	2.336	–	84.28	39.78	56
	3.708			45.51	
	3.709			45.51	
	3.708			59.74	
	3.709			339.99	
	2.234			364.93	

**Figure 7:**
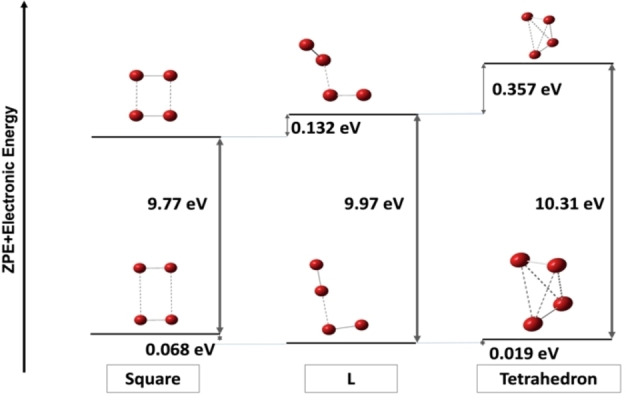
Energy level diagram of three considered Br_4_ conformers. Geometry optimizations and frequency calculations are performed at camB3LYP/aug‐cc‐pVTZ for Square and L, with the energies taken as the sum of the CCSD(T)/aug‐cc‐pVTZ electronic energy and ZPE calculated at the DFT level. Geometry optimizations and frequency calculations are performed at M062X/aug‐cc‐pVTZ for L and Tetrahedron, with the energies taken as the sum of the CCSD(T)/aug‐cc‐pVTZ electronic energy and ZPE calculated at the DFT level. Note that the relative energetics for the L structure are identical across DFT methodologies at the reported precision.

For each isomer considered, the adiabatic ionization energy is refined with CCSD(T) single point calculations. While the use of different functionals typically excludes direct comparison, the fact that the L‐isomer is identically captured by both DFT levels allows for relative energetics to be qualitatively useful. Indeed, starting from the M06‐2X optimized L‐isomer, the single‐point calculations find it is higher in both the case of the neutral and cation than the corresponding camB3LYP version by less than 2 meV, leading to an identical AIE also within 2 meV. A schematic energy level diagram is given in Figure 7.

Based on the sole existing claim of Br_4_^+^ as a polar ion in the gas phase (albeit with no other structural information), one is inclined to assume that the polar, twisted “L” structure is the dominant Br_4_^+^ conformer.^[8]^ Contrastingly, based on analogy with crystalline Cl_4_^+^ (and the dimerization of two I_2_^+^ ions, I_4_^2+^), the rectangular structures similarly appear likely.[^3a^, ^6b^] However, based on the calculated CCSD(T)/aug‐cc‐pVTZ AIE for each candidate alone, only the tetrahedron with an AIE of 10.31 eV is near the onset of the experimental spectrum. Both the tetrahedron and L‐shaped neutral Br_4_ are expected to be more thermodynamically favored than the square neutral by approximately 50 and 70 meV, respectively. Simulations are integrated and overlayed with the observed photoionization spectrum in Figure 6. Notably, the significant geometry difference of one Br_2_ rotating out of plane in the L cation← neutral transition results in a near‐zero overlap for the vibrational wavefunctions, a feature that is agnostic to the level of DFT employed and renders the simulation unreliable. This consequence extends to transitions between isomer pairs, and we predict no observable vertical transitions in mixed isomer pairs. While the square transition matches qualitatively in shape as a sharp rise to plateau, an unphysical energy shift of approximately +0.5 eV needs to be applied in order to find agreement with the observed PI spectrum. No significant shift is required in the case of the elongated tetrahedron. This close match in calculated AIE at the CCSD(T) level with the strong agreement in the shape of the spectrum provides evidence that the observed ion signal originates from a thermodynamically favorable tetrahedron‐shaped Br_4_ molecule formed as a recombination product during bromoform dissociation.

## Conclusions

In summary, the Br_4_^+^ ion is detected upon the photodissociation of bromoform using photoionization mass spectrometry coupled to tunable synchrotron radiation. This allows for a joint theoretical and experimental investigation resulting in the proposed structural characterization of Br_4_ and Br_4_^+^. Optimized bond parameters and vibrational frequencies are computationally determined with the coulomb‐attenuating DFT method cam‐B3LYP/aug‐cc‐pVTZ or M06‐2X/aug‐cc‐pVTZ, and single point energetics are found with the *ab‐initio* CCSD(T)/aug‐cc‐pVTZ level of theory in all cases. Simulating Franck‐Condon overlap integrals allows for a theoretical photoelectron spectrum to be integrated and compared against the experimental photoionization spectrum. Results point to the formation of Br_4_ in the gas‐phase to have a distorted tetrahedral geometry of *D_2h_* symmetry. Insights into the mechanism of Br_4_ synthesis following initial bromoform photolysis in the gas phase is still unknown and a subject of further investigation.

## Conflict of interests

The authors declare no conflict of interest.

1

## Data Availability

The data that support the findings of this study are available from the corresponding author upon reasonable request.
